# Inter-subject correlation of audience facial expressions predicts audience engagement during theatrical performances

**DOI:** 10.1016/j.isci.2024.109843

**Published:** 2024-04-29

**Authors:** Richard A. Oakes, Lisa Peschel, Nick E. Barraclough

**Affiliations:** 1 Department of Psychology, University of York, Heslington, YO10 5DD York, UK; 2 Department of Theatre, Film and Television and Interactive Media, University of York, Heslington, YO10 5DD York, UK

**Keywords:** Artificial intelligence, Psychology, Social sciences

## Abstract

During performances, audiences experience various emotional states, and these are reflected in their ongoing facial expressions. We investigated if audience engagement could be determined by measuring the inter-subject correlation (ISC) of non-invasively recorded audience facial expressions. We filmed the faces of multiple audience members at theatrical performances and determined the intensity of their different facial expressions throughout the performances. Neutral, happy, anger, and disgust expression ISCs accounted for up to 24% of the performance dramaturge’s predictions of audience engagement. Expression synchrony was greater between individuals in close proximity, suggesting effects of emotional contagion or cognitive similarities between neighboring individuals, whereas expression synchrony was greatest between individuals who were younger, female, and with greater levels of empathy, showing that individual characteristics impact shared audience experiences. Together, our results show that facial expression synchronization could be used as a real-time non-invasive indicator of engagement in audiences larger than achieved using previous approaches.

## Highlights

• During performances audience facial expression synchrony predicts their engagement
• Expression synchrony increases with spatial proximity within the auditorium
• Younger, female, and more empathetic individuals show greater expression synchrony
• Videoing faces of large groups allows real-time non-invasive measures of engagement

## Graphical abstract

**Figure:**
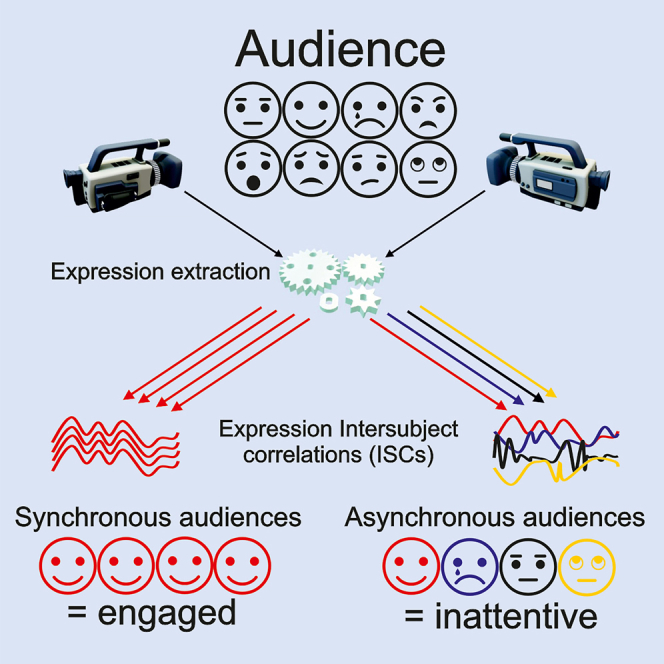

## Inter-subject correlation of emotional expressions in audiences during theatrical performances

Measuring the facial expressions of members of an audience has recently helped our understanding of their experiences when watching artistic performances. Emotional experiences are one of the key reasons people attend artistic performances and are quantitatively different when experienced live and within an audience group.^1^^,^^2^^,^^3^ During artistic performances individuals in the audience experience various emotional states, and different emotions are often reflected in specific changes in the facial expressions of the individual.^4^^,^^5^^,^^6^ Information from facial expressions during the performance can enable an understanding of the emotional experience of the individual audience members complementary to that obtained by self-reports.^7^ In this prior study, we filmed the faces of the audiences seated in an auditorium while they listened to a musical concert and subsequently determined their different facial expressions using face expression analysis software. Pieces of music that expressed sadness resulted in more facial expressions of sadness, whereas pieces of music that expressed happiness resulted in more facial expressions of happiness. This close relationship between audience facial expressions during the performance and their subsequent reports of the emotional experiences they experienced permits a non-invasive method of determining how audiences may experience artistic performances.

The evaluation of the ongoing facial expressions of audience members provides information about audience experiences not possible with other methods. Traditionally, audience experiences of artistic performances have been studied using self-reports.^3^^,^^8^^,^^9^ Self-reports, however, are potentially open to bias and only allow a retrospective analysis of emotional experiences. In contrast, measuring the expressions produced by audience members has the potential to provide time-locked moment-by-moment information about emotions experienced during long performances. Although ongoing emotional experiences have previously also been recorded using physiological measures of skin conductance^10^ or facial electromyography,^11^ these methods have drawbacks. The attachment of electrodes to audience members is time-consuming and invasive and thus impacts the audience experience of the performance. In addition, this becomes unfeasible in larger audiences as the number of individuals that can be studied is restricted by the availability of equipment. In contrast, high temporal resolution measurements of dynamic changes in facial expressions of large audiences are possible using unobtrusive filming of faces, allowing estimates of emotional experiences within ecologically valid environments.^7^

In addition to information about the emotions of audience members, facial expressions may also provide information about audience engagement. Audience engagement is a multifaceted concept that has traditionally been difficult to measure, as discussed in multiple reports.^9^^,^^12^^,^^13^^,^^14^ It is thought to comprise affective responses, attention, cognitive interactions, empathy processes, and social, cultural, and contextual influences. However, during periods of high engagement with the performance, it is thought that audience members enter a form of “flow”^15^ where they “get lost” in the performance or are captivated by it. This state of captivation is not dependent upon high degrees of specific emotions, but rather the consistency of how the performance affects the emotional state of the members of the audience. Consequently, the degree of emotional synchronization within the audience as a whole, which reflects how consistently the performance influences the emotional states of all audience members, could potentially be a useful measure of audience engagement. High states of emotional synchrony where there is increased behavioral “alignment”^16^ would occur during high audience engagement with the flow of the narrative and emotional content of the performance. In contrast, lower emotional synchrony would occur when the audience members were not attending, having their mind wandering or thinking about topics other than the performance.

Indeed, studies have recently shown that the degree of audience engagement is reflected in the synchrony between a range of different physiological^17^^,^^18^^,^^19^^,^^20^ and neural^21^^,^^22^^,^^23^ signals of audience members. These methods were originally developed to analyze the reliability of responses of multiple individuals during the multidimensional dynamics of real-world social contexts.^24^^,^^25^^,^^26^ Here, the inter-subject correlation (ISC) between individuals provides a measure of shared experiences of complex naturalistic experiences that unfold over many minutes. ISCs can show how differently we synchronize or “tick together,” during emotional experiences,^27^ and variance in ISCs reflects differences in experiences between individuals with different psychological characteristics.^28^ Consequently, the degree of ISC of a range of different measured signals is related to the degree of audience engagement.^29^ For example, synchronization in audience heart rates is related to self-reported narrative engagement when viewing television and film content.^19^ Furthermore, during music listening, high ISCs of facial muscles, cardiorespiratory, and skin conductance responses were consistently related to periods of arousing emotions and audience engagement.^18^ Whereas peak correlations in neural activity measured using electroencephalography (EEG) correspond also with arousing moments during film viewing and reflect attention- and emotion-modulated processing.^30^ And large-scale audience engagement with, and preference for, television adverts can be predicted by peaks in neural synchrony measured in a different and smaller sample of viewers.^22^ Thus, ISCs of a range of different signals have all been shown to be good measures of audience engagement.

Given these findings, in this study, we investigated if audience engagement could be determined by measuring the ISC of non-invasively recorded audience facial expressions. Prior research has relied on intrusive methods of measuring audience responses requiring the placement of multiple electrodes on the face, scalp, or torso and have been restricted to relatively small (*n* < 16) audiences. Measuring audience facial expression ISC synchrony has the potential to allow us to assess audience engagement in much larger groups of individuals while also limiting intrusive measures that detract from the audiences’ experience of the artistic performances. Therefore, in this study, we tested if this was possible by videoing the faces of multiple audience members at several different theatrical and cabaret performances. In order to ensure the viability of this method “in the wild,” we ensured the performances were as naturalistic as possible, with actors performing in public auditoriums while audience members were filmed with unobtrusive cameras. We tested our hypothesis that facial expression ISCs would be related to predictions of audience engagement made by the performance “dramaturge” (the expert in the piece of work performed who advised the director and other collaborating artists on the production). Strong evidence of such a relationship would indicate the potential utility for this method to determine audience engagement non-invasively in the future.

There are also other possible sources of behavioral alignment between different individuals during a performance at a theater,^16^ including between the actors on stage, between actors and the audience, between individual audience members in the auditorium, or across the audience more broadly. During such shared experiences individuals become “aligned”^31^ physiologically, mentally, and in their expression of emotions in their facial movements. There is much research on face-to-face dyadic interactions that have shown that physiological measures^32^^,^^33^^,^^34^ and facial expressions^35^^,^^36^^,^^37^ are often synchronized or highly similar, resulting from a continuous mutual adaptation between the two individuals. This type of “dynamic coupling”^31^ where one individual is interacting with another individual and each are reacting to the facial expressions of the other is unlikely to contribute significantly to any putative audience expression synchrony. During performances, audience members are not typically interacting directly with each other, instead they will be spending more time facing toward the stage and attending the performance (as confirmed by our recording). Any such interactions will likely be limited to pre- and post-performance discussions between neighboring individuals who are familiar with each other, or at least during performances these interactions will be fleeting. Actor-actor interactions are not the focus of our study, and our methods cannot track their expressions as they move around on stage. Instead, the interaction of interest is that between each audience member and the “performance” by the actor(s) as a collective, which is a function of the degree of audience engagement in the performance.

However, even if audience members do not interact intentionally, they can have a degree of synchrony in their emotional responses due to their shared experience.^38^ Pairs of individuals watching a movie together show synchronization of autonomic responses^39^^,^^40^ and facial expressions,^40^^,^^41^^,^^42^ which are closely linked.^43^^,^^44^ Putative mechanisms by which this occur may involve mimicry,^45^ a tendency to automatically synchronize affective expressions of another person, or emotional contagion, a related concept where we “catch” other people’s emotion.^46^^,^^47^ The strongest effects of such emotional synchronization are likely to occur between individuals who are closest to each other in the auditorium for two reasons. First, individuals in closer proximity will be more sensitive to the small changes in the behavior of their immediate neighbors and experience emotional contagion, e.g., see^39^^,^^41^^,^^46^^,^^47^^,^^48^. Second, individuals who attend a performance together will likely be sitting in seats next to, or nearby each other, and may share some similar interests and personal characteristics. Such social similarities may result in increased behavioral and emotional alignment between these individuals. Therefore, we tested the hypothesis that the spatial proximity of individuals within a large audience influences the degree of measured facial expression synchrony.

In addition, it has been shown that the characteristics of individuals can impact ISC of both physiological^49^^,^^50^^,^^51^^,^^52^^,^^53^ and neural^21^^,^^27^ signals. For example, skin conductance synchrony between patients and therapists can be related to perceptions of perceived empathy.^50^^,^^51^ Whereas the ISC of neural activity within the posterior middle temporal gyrus (MTG), a region of cortex associated with reading intentions of other individuals and more generally with empathy and mentalizing,^54^^,^^55^ is positively associated with self-reported emotional empathy scores.^27^ In addition, individual empathy has also been shown to predict synchrony in small groups of students within the classroom.^21^ Given this prior research in both dyads and groups, we predicted that the empathy of those individuals within large audiences at theatrical performances would impact the degree to which their expressions were synchronized. We hypothesized that pairs of individuals in the audience showing higher degrees of empathy would show a greater degree of facial expression ISCs.

Finally, the age and the gender of individuals have been shown to also impact ISC in some cases. Older participants can have reduced ISC when measured using functional magnetic resonance imaging (fMRI)^56^^,^^57^ or EEG,^58^^,^^59^ maybe because idiosyncratic brain activity arises with age, leading to increased neural variance and thus a decrease in ISC. We therefore tested whether the age of individuals influenced face expression ISCs and hypothesized that expression ISC would decrease with age.

The impact of gender on ISC is more complex, however. In children, but not adults, greater EEG ISC is seen in males than females.^58^ This may reflect that in younger individuals males have comparably reduced brain development than females of the same age,^60^ whereas comparatively advanced brain development in the equivalently aged young females results in greater neural variance and thus lower EEG ISCs. Others, however, have observed higher ISC in males compared to females in some brain regions (e.g., posterior and central midline), but lower ISCs in males in other brain regions, e.g., temporal parietal junction and inferior temporal cortex.^56^ Given this mixed picture, we could not make strong predictions about the impact of gender of audience members on facial expression ISCs, but instead explored the impact of gender in younger and older individuals within the audience.

In summary, in this paper we report several investigations into the synchrony of facial expressions within large audiences attending theatrical performances. We first describe the extent of the synchrony between a number of different emotional expressions that we could derive from video footage of audience faces. We then test the hypothesis that audience engagement can be determined by measuring the ISC of audience facial expressions. The aim here was to assess the potential utility for this method to determine audience engagement non-invasively. We additionally test whether increased spatial proximity of audience members increases expression synchrony. Finally, we assess the influence of audience member empathy, age, and gender on expression synchrony.

## Results

For the 193 audience members who met our criteria for inclusion, we calculated the mean magnitude of each expression, and the standard deviation of each expression, throughout the entirety of the performance for each individual (see Table 1). These values show the degree to which each expression is on average present during the performances and how much these expressions vary during the performances. The neutral expression was detected most commonly, as indicated by a large mean magnitude, whereas the emotional expressions were detected less. The happy, sad, and anger expressions were the most commonly present emotional expressions. There was considerable variance in expressions during the performances, in particular the neutral, happy, sad, and anger expressions. There were no significant differences in mean or variance of expressions between female or male individuals.

**Table 1:** Average magnitudes and variances in expressions during performances

Expression	Magnitude	Standard deviation
Neutral	0.68 (0.16)	0.16 (0.05)
Happy	0.10 (0.12)	0.14 (0.09)
Sad	0.08 (0.08)	0.10 (0.07)
Anger	0.08 (0.12)	0.10 (0.10)
Surprise	0.01 (0.02)	0.03 (0.04)
Fear	0.02 (0.04)	0.04 (0.04)
Disgust	0.02 (0.05)	0.03 (0.05)
Contempt	0.065 (0.06)	0.09 (0.07)

Note: Expression magnitude varies between 0 and 1; the value in brackets is the standard deviation in these values across participants. Standard deviation refers to average variance in the expression over the duration of the performance; the value in brackets is the standard deviation in these values across participants.

To determine whether FaceReader treated the seven different emotional expressions, and the neutral expression, as mutually exclusive or not, for each individual we correlated these eight signals recorded during the performance. The average r values (see Table 2) indicated a substantial degree of independence of these expressions, reflecting FaceReader’s method of classification of expressions based upon face action unit activity. Neutral expressions were moderately negatively correlated with the happy expressions, but only weakly negatively correlated with the other expressions. All other expressions were only very weakly correlated with each other.

**Table 2:** Mean (standard deviation) between-expression correlations for all participants

	Neutral	Happy	Sad	Anger	Surprise	Fear	Disgust
Happy	−0.56 (0.33)						
Sad	−0.20 (0.31)	−0.17 (0.15)					
Anger	−0.18 (0.32)	−0.12 (0.16)	−0.04 (0.17)				
Surprise	−0.01 (0.18)	0.03 (0.15)	−0.05 (0.14)	−0.05 (0.13)			
Fear	−0.06 (0.18)	0.00 (0.16)	−0.00 (0.15)	−0.00 (0.16)	0.17 (0.18)		
Disgust	−0.14 (0.20)	0.07 (0.18)	0.03 (0.20)	−0.02 (0.17)	0.05 (0.14)	0.12 (0.19)	
Contempt	0.14 (0.15)	−0.03 (0.15)	−0.01 (0.13)	−0.05 (0.11)	−0.01 (0.11)	−0.02 (0.11)	−0.03 (0.12)

### Facial expressions are correlated within audiences

In order to understand how an individual’s expressions were related to those produced by the rest of the audience, we examined the correlations between all eight expressions measured. The eight expressions produced during the whole performance for each individual were correlated with the averages of the eight expressions from the rest of the audience (audience n-1).

**Figure 1:**
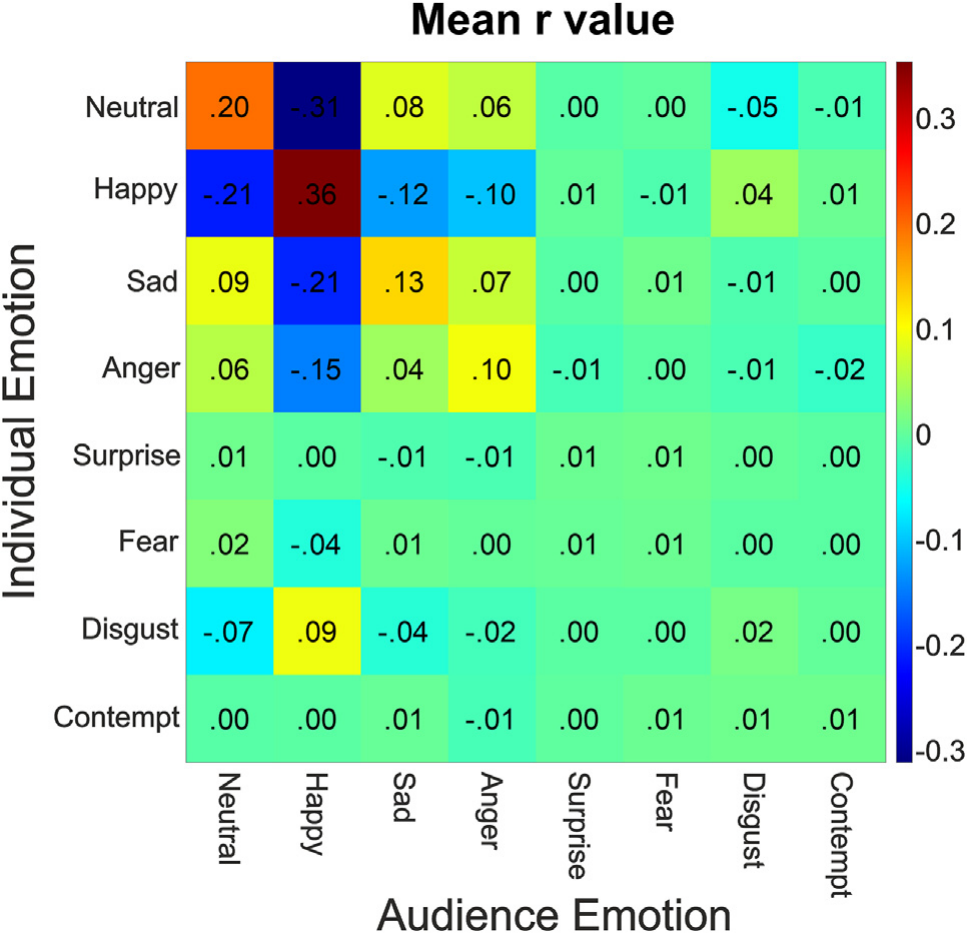
Relationship between expressions of each audience member and the average expressions calculated across the rest of the audience Expressions of the individuals are on the y axis, expressions of the rest of the audience are on the x axis. Redder colors indicate larger positive r values, and bluer colors indicate larger negative r values; mean transformed r values are indicated within each cell.

To understand how audience expressions are correlated with each other across all 16 performances, we calculated the mean r values for expression correlations from all 193 similarity matrixes and present this as a single similarity matrix in Figure 1. Here, a larger positive mean R value indicates that for a particular expression in individuals there is a positive correlation with a particular expression in the rest of the audience as a whole (i.e., for those expressions, audience expressions are in synchrony). Negative mean r values, in contrast, indicate that for a particular expression in individuals there is negative correlation with a particular expression in the rest of the audience as a whole (i.e., for those expressions, the audience expressions are asynchronous).

As might be expected given that each member of an audience experiences the same performance, expressions are largely correlated within-expression. For example, the level of happiness expressed by one individual appears associated with the level of happiness expressed in the rest of the audience as a whole. This suggests a degree of shared emotional experience across audience members. Of all the expressions measured, happy expressions appear most correlated with each other, and these appear negatively correlated with non-happy expressions, in particular neutral, sad, and angry expressions. To assess the degree to which synchrony is within-expression, we compared mean within-expression (on-diagonal) and mean between-expression (off-diagonal) correlation values with a Bayesian paired-samples t test. Our null hypothesis was that there was no difference between within-expression correlations and between-expression correlations. Our alternative hypothesis was that within-expression correlations would be greater than between-expression correlations. The Bayes factor (BF_10_ = 1.33x10^52^) indicated extreme evidence for our alternative hypothesis that within-expression correlations were greater than between-expression correlations. The error % could not be calculated as the BF_10_ value was so large; however, the median of the resulting median posterior distribution was 1.60, with a central 95% credible interval that ranged from 1.39 to 1.81. Thus, as audience expressions are more correlated within-expression, it suggests the emotions of individuals within an audience tend to be in synchrony with each other.

We then assessed how well each of the different facial expressions were correlated with themselves compared with their correlations with the other expressions. We did this by first averaging the seven between-expression (off-diagonal) correlations for each expression separately and then compared these values to the respective within-expression (on-diagonal) correlation with separate Bayesian paired-samples t tests. The Bayes factors (Table 3) indicated extreme evidence for our alternative hypothesis that within-expression correlations were greater than between-expression correlations for the majority of expressions including neutral, happy, sad, angry, and disgust expressions and anecdotal evidence for a difference for surprise and contempt expressions. Whereas Bayes factors indicated anecdotal evidence that fear within-expression correlations were not different from fear between-expression correlations.

**Table 3:** Within-expression correlations vs. between-expression correlations

Expression	BF_10_	Error %	Median posterior distribution	Central 95% credible interval
Neutral	5.72 × 10^27^	NaN	0.987	[0.815, 1.161]
Happy	3.02 × 10^54^	NaN	1.662	[1.441, 1.882]
Sad	2.71 × 10^26^	NaN	0.956	[0.785, 1.127]
Angry	1.35 × 10^21^	NaN	0.798	[0.827, 0.992]
Surprise	2.46	0.0004	0.169	[0.037, 0.309]
Fear	0.45	<0.0001	0.112	[0.011, 0.249]
Disgust	199	<0.0001	274	[0.132, 0.417]
Contempt	1.36	0.0004	0.150	[0.026, 0.029]

### Facial expression inter-subject correlations are related to predictions of audience engagement

Expression ISCs varied considerably over the duration of a performance and were independent of the absolute magnitude of the expressions themselves (see Figure S1; Table S1). Audience expression synchronization could be considerable at points, for example with peaks in correlation of happy expressions greater than r = 0.4 during a performance in York in 2017. On average, happy expressions were the most synchronized (mean r across performances = 0.070, SD = 0.004, max = 0.416). Indeed, happy expressions were typically over twice as synchronized as the neutral expressions and over four times as synchronized as sad expressions (see Table 4). What is most evident is that there is considerable variation in the ISCs over the duration of the performances, indicating that while audiences freely viewed complex multimodal theatrical performances there are notable periods when the emotional state of audiences appear to “tick together.”^61^

**Table 4:** ISCs for three performances

Expression	Maximum r	Mean r
Neutral	0.17	0.03
Happy	0.42	0.07
Sad	0.09	0.02
Anger	0.09	0.02
Surprise	0.05	0.01
Fear	0.05	0.00
Disgust	0.05	0.01
Contempt	0.04	0.01

**Figure 2:**
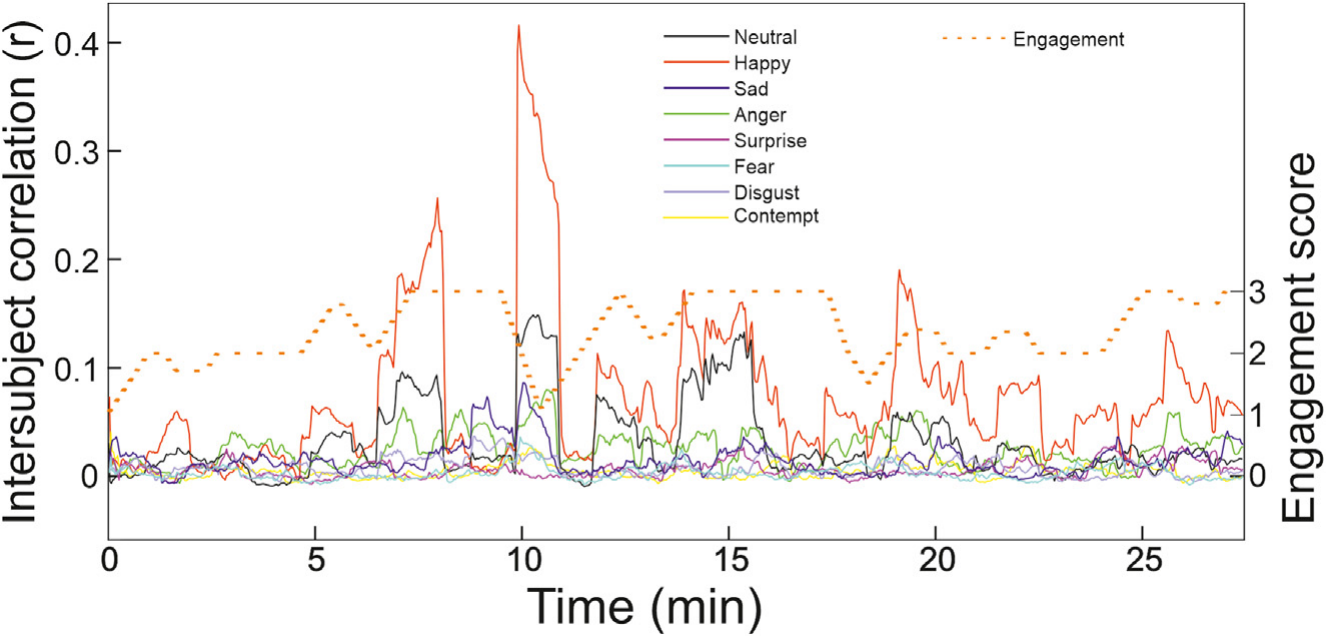
Inter-audience expression synchrony is related to predictions of audience engagement Example from a single performance at York in 2017. Expression synchrony is illustrated as raw waveforms representing fluctuations in ISC r values for each expression over the duration of the performance (solid lines). Prediction of audience engagement on a 1–3 scale at each point in the performance is also illustrated as a waveform (dotted orange line).

A comparison between the expression ISCs and the dramaturge’s predictions of audience engagement across three different performances (Figure 2) showed that the neutral, happy, anger, and disgust expression ISCs were consistently and positively correlated with estimated audience engagement across the three performances (see Table 5). Bayes factors indicated extreme evidence for a positive relationship between engagement and neutral, happy and disgust expression ISCs in all three performances, and for angry expression in two performances, with moderate evidence for angry expressions in one performance. Bayes factors indicated very strong evidence for sadness, fear, and contempt expression ISCs being positively correlated with audience engagement in one performance. Finally, the Bayes factor indicated extreme evidence for surprise expression ISCs being negatively correlated with audience engagement in one performance.

**Table 5:** Bayes factors for correlations between facial expression ISCs and engagement for performances P1–P3

Expression	P1		P2		P3	
	r^2^	BF_10_	r^2^	BF_10_	r^2^	P3 BF_10_
Neutral	0.35	1.05 × 10^25^	0.22	2.39 × 10^7^	0.40	3.84 × 10^30^
Happy	0.36	7.70 × 10^26^	0.29	3.10 × 10^14^	0.30	2.88 × 10^16^
Sad	0.07	0.41	0.26	5.06 × 10^11^	0.10	4.51
Angry	0.25	1.85 × 10^11^	0.36	5.91 × 10^24^	0.11	8.99
Surprise	−0.14	466.62	−0.05	0.14	.-05	0.12
Fear	0.07	0.36	0.23	3.51 × 10^8^	−0.04	0.07
Disgust	0.15	1667.67	0.28	1.24 × 10^13^	0.24	4.37 × 10^9^
Contempt	−0.06	0.24	0.10	2.23	0.13	41.49

We then calculated three separate (one for each performance) Bayesian linear regression models to examine the predictive value of expression ISCs on the estimate of audience engagement. For all three performances, following observation of the data, the odds in favor of models where expression ISCs predicted audience engagement increased. Optimal models are described in Table 6; however, the odds in favor of many other models where different combinations of expression ISCs predicted audience engagement also increased, but for brevity are not reported here. For the optimal models, Bayes factors indicated very strong evidence that expression ISCs predicted audience engagement, and they accounted for 24%, 19%, and 22% of the variance in the three performances.

**Table 6:** Optimal Bayesian regression models for expression ISCs predicting audience engagement

Performance	Optimal model	Optimal model BF_M_	Optimal model R^2^
P1	Neutral, Happy, Anger, Surprise, Fear, Disgust	140.82	0.24
P2	Anger, Fear, Disgust	57.64	0.19
P3	Neutral, Surprise, Fear, Disgust, Contempt	129.05	0.22

### ISCs are influenced by audience member proximity

**Figure 3:**
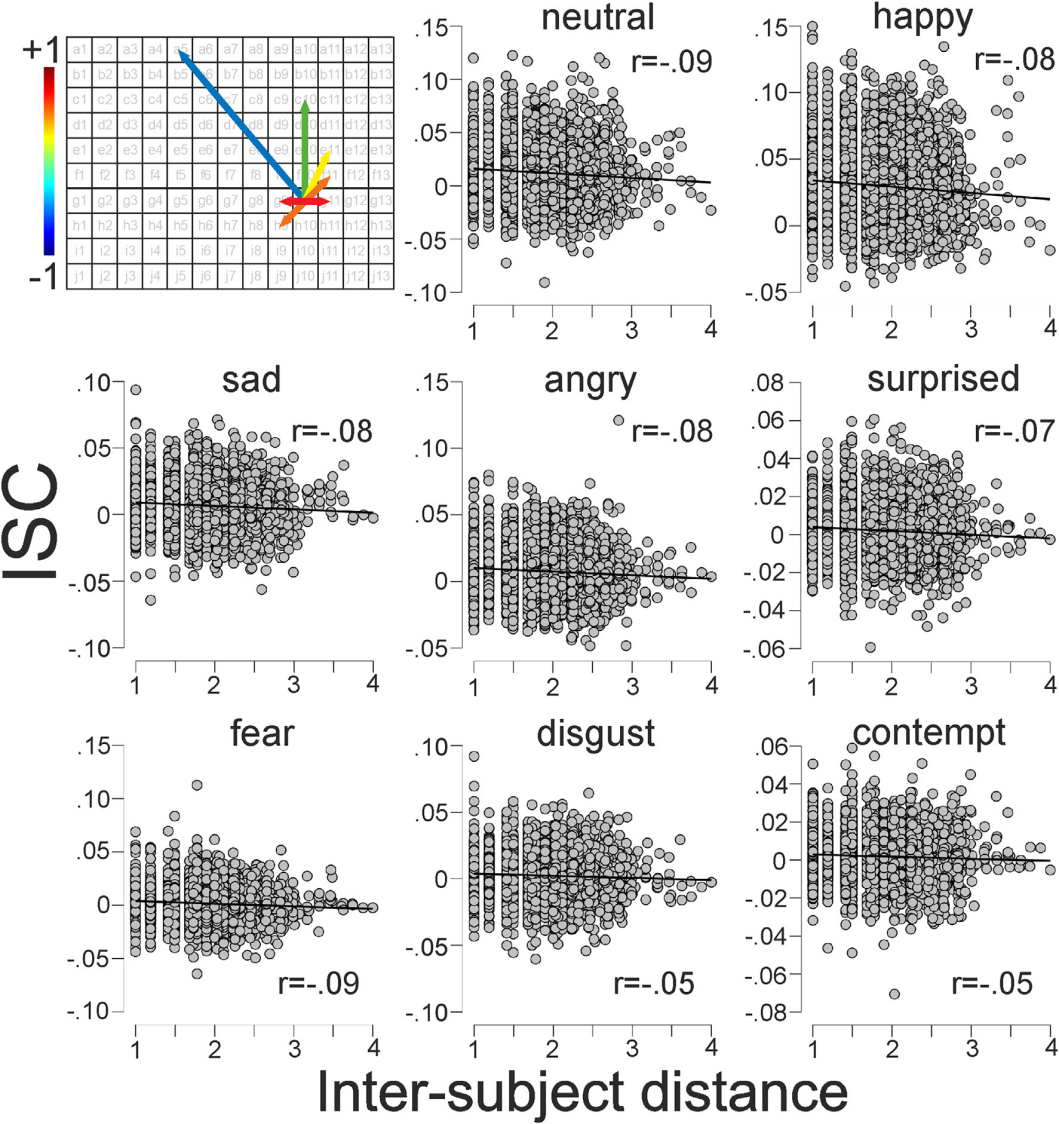
ISCs are related to audience proximity Upper left panel illustrates a grid representing the arrangement of seats in a hypothetical auditorium. For a given audience member (e.g., sitting in row g seat 10), the distance between them and each of the other audience members was calculated and then correlated with the degree of synchrony between each pair of individuals (other panels). The color of the arrows representing these relationships illustrate a hypothetical situation where audience proximity is related to emotional synchrony (redder colors = higher correlations, bluer colors = little to no correlations). Other panels: correlations (including r values) between transformed audience ISCs and the square root of the spatial proximity in the auditoriums.

In line with our prediction, there were small negative correlations between the distances between audience members and expression ISCs, indicating that the further apart audience members were from each other, the less their expressions were synchronized (see Figure 3). Bayes factors indicated extreme evidence for a negative relationship between audience member proximity and neutral (BF_10_ = 11088), happy (BF_10_ = 2317), sad (BF_10_ = 916), angry (BF_10_ = 498), surprise (BF_10_ = 276), and fear (BF_10_ = 6665) expressions and anecdotal evidence for this relationship with disgust (BF_10_ = 2.68) and contempt expression ISCs (BF_10_ = 1.18). A permutation test (see Table S2) indicated that all r values shown in Figure 3 were robust.

To test whether audience member spatial proximity predicted the different expression ISCs, we calculated eight separate Bayesian linear regression models. Following observation of the data, the odds in favor of models where audience member proximity predicted facial expression ISCs (BF_M_) increased for all expressions (see Table 7).

**Table 7:** Bayesian regression models testing if proximity predicts ISCs

Emotion	BF_M_	Model R^2^	Mean	SD	95% credible interval
Neutral	18,985	0.01	−0.004	<0.001	[−0.006, −0.003]
Happy	3,988	0.01	−0.005	0.001	[−0.007, −0.003]
Sad	1,582	0.01	−0.003	<0.001	[−0.005, −0.001]
Angry	862	0.01	−0.003	<0.001	[−0.003, −0.002]
Surprise	479	0.01	−0.002	<0.001	[−0.003, −0.001]
Fear	11,431	0.01	−0.002	<0.001	[−0.003, −0.001]
Disgust	4.73	0.00	−0.001	0.001	[−0.002, −0.000]
Contempt	2.08	0.00	−0.000	<0.001	[−0.002, −0.000]

### ISCs are dependent upon audience member age, gender, and level of empathy

We tested the impact of audience member age and level of empathy on facial expression ISCs. We predicted that ISCs would decline with the age of audience pairs, but increase with audience pair empathy. For each audience pair we calculated the combined (total) age as well as the combined (total) empathy score. Bayesian correlations between expression ISCs and total age and total empathy are described in Table 8. For all expressions age was negatively correlated with ISCs. Bayes factors indicated extreme evidence for a negative relationship between age and neutral, happy and angry ISCs, and anecdotal evidence for a negative relationship with fear and disgust ISCs. Whereas there was anecdotal evidence for no relationship between age and both sad and contempt ISCs and strong evidence for no relationship with surprise ISCs.

**Table 8:** Correlations between facial expression ISCs and audience age and empathy

Emotion	Age		Empathy	
	r^2^	BF_10_	r^2^	BF_10_
Neutral	−0.12	1.02 × 10^9^	0.08	605.64
Happy	−0.19	1.18 × 10^27^	0.08	1012.55
Sad	−0.04	0.40	−0.01	0.03
Angry	−0.13	9.51 × 10^10^	0.07	52.18
Surprise	−0.01	0.03	0.01	0.03
Fear	−0.05	1.18	−0.00	0.02
Disgust	−0.05	1.32	0.03	0.10
Contempt	−0.04	0.20	0.01	0.02

In contrast to age, audience empathy appeared positively correlated with most expressions ISCs, apart from sad and fear ISCs. Bayes factors indicated extreme evidence for a positive relationship between empathy and neutral and happy ISCs and very strong evidence for a positive relationship with angry ISCs. However, there was moderate evidence for no relationship between empathy and disgust ISCs and very strong evidence for no relationship with sad, surprise, fear, and contempt ISCs.

We then calculated eight separate Bayesian linear regression models to examine the predictive value of the age and empathy scores on the magnitude of each of the expression ISCs. Following observation of the data, the odds in favor of models where both audience age and empathy predicted expression ISCs increased for neutral, happy, and angry expressions (see Table 9). The inclusion Bayes factors indicated that for these expressions age is an extremely strong predictor of decreasing ISCs, but empathy also has a slightly smaller, but very strong predictive role of increasing ISCs. In addition, following observation of the data, the odds in favor of only age predicting ISCs increased for fear and disgust expressions. The inclusion Bayes factors indicated that for these expressions there was anecdotal evidence of age predicting decreasing ISCs, whereas strong or anecdotal evidence that empathy did not predict fear or disgust ISCs, respectively. Finally, following observation of the data, the odds in favor of neither age nor empathy predicting sad, surprise, and contempt expressions (the null model) increased.

**Table 9:** Bayesian regression models testing if age or empathy predicts ISCs

Emotion	Optimal model	Optimal BF_M_
Neutral	Age & Empathy	2,275
Happy	Age & Empathy	2,565
Sad	Null model	3.70
Angry	Age & Empathy	203
Surprise	Null model	28.69
Fear	Age	5.37
Disgust	Age	4.10
Contempt	Null model	7.00

To explore the impact of gender on expression ISCs, for each audience pair we calculated whether the pair were of the same gender where both were male, or both were female, or whether the audience pair were of different genders. Given that some prior research has indicated that gender differences in ISCs can be dependent upon age,^58^ we also calculated whether the audience pairs were young (both <25 years old) or old (both >25 years old) or whether the audience pair were of different ages. We then analyzed how the magnitudes of different expression ISCs were dependent upon gender and age with eight separate Bayesian ANOVA models. We compared both a model with gender as a main effect and a model including both the main effect of gender and the interaction between gender and age, against a null model that also included the main effect of age (as the effect of age had already been explored earlier).

**Figure 4:**
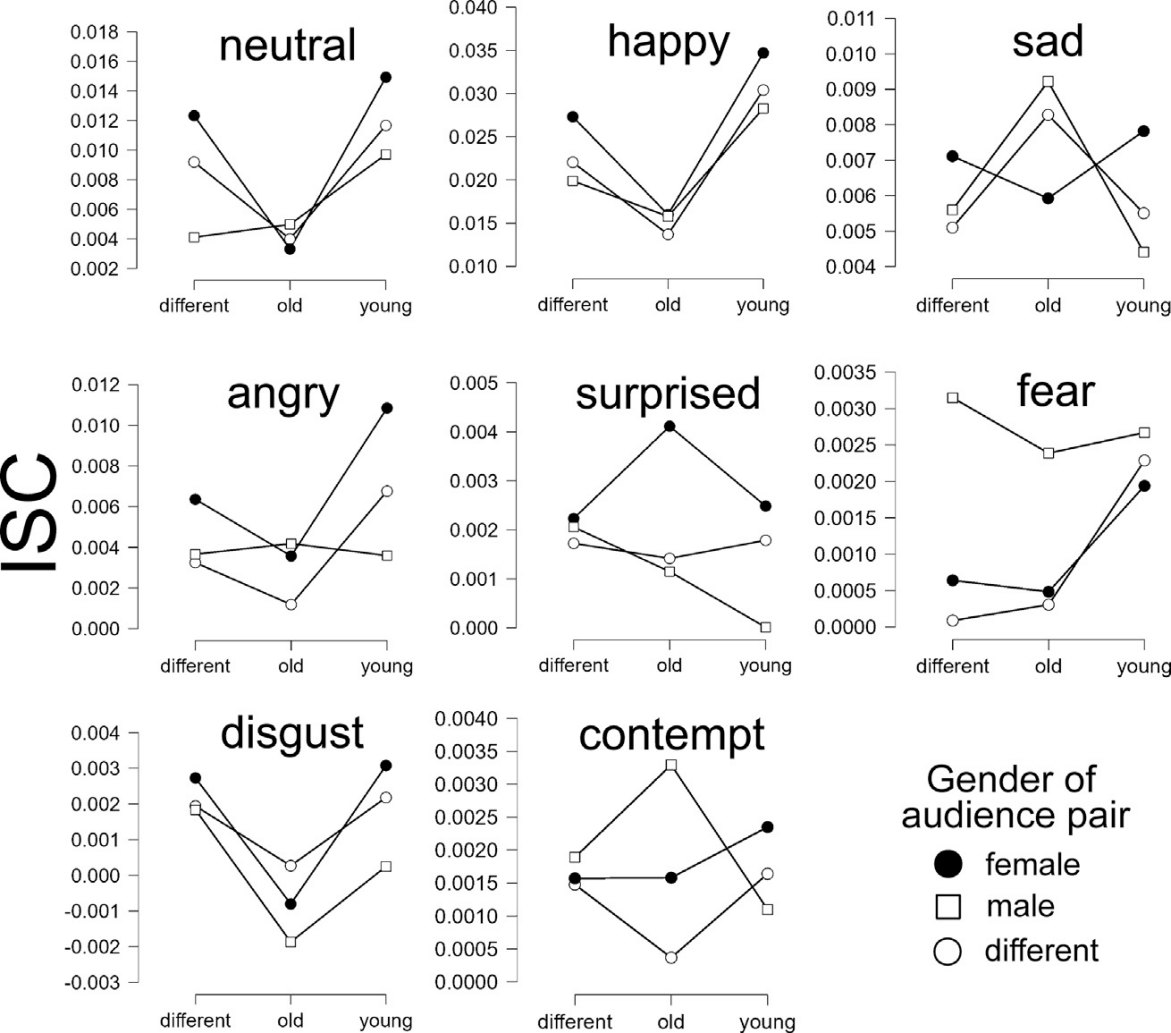
The influence of gender on ISCs Separate panels indicate the mean transformed ISCs for the eight expressions. Mean ISCs are calculated between pairs of individuals grouped according to gender: female (both female), male (both male), different (male and female), and according to age: young (both <25 years old), old (both >25 years old), different (one individual <25 and one individual >25).

Following observation of the data, there was strong or greater evidence that ISCs were different across genders for neutral (BF_M_ = 63.00), happy (BF_M_ = 455.01), and angry (BF_M_ = 104.20) expressions (see Figure 4). For these expressions, ISCs between females were greater than ISCs between males. In contrast, for fear (BF_M_ = 0.03) and contempt (BF_M_ = 0.03) expressions there was strong evidence that ISCs were similar across genders. For the other expressions, there was only moderate or anecdotal evidence that gender mattered for sad expressions (BF_M_ = 8.46) or did not matter for surprise (BF_M_ = 0.12) or disgust (BF_M_ = 0.13) expressions.

The model that included the interaction between gender and age was compared against all other models, allowing us to examine whether gender differences in ISCs were dependent upon age. Following observation of the data, there was strong or greater evidence that there was no interaction between gender and age for neutral (BF_M_ = 0.01), happy (BF_M_ = 0.01), sad (BF_M_ = 0.02), angry (BF_M_ = 0.04), surprise (BF_M_ = 4.45 x 10^−4^), fear (BF_M_ = 9.40 x 10^−5^), disgust (BF_M_ = 3.65 x 10^−4^), and contempt (BF_M_ = 1.34 x 10^−4^) expressions. In summary, there was no evidence that gender differences in facial expressions ISCs were different between individuals of different ages.

## Discussion

Across a range of different theatrical and cabaret performances we filmed the expressions conveyed by large numbers of audience members as the performances unfolded. These measurements allowed us to assess the intensity of neutral, happy, sad, angry, surprise, fear, disgust, and contempt expressions every 20 min in each face throughout the duration of the performances. We evaluated the degree of expression synchrony by measuring the correlations between expression intensity functions. We found that the facial expressions of audience members showed varying degrees of synchrony between the different expressions and varying synchrony over time. The greatest degree of audience synchrony was seen within-expression particularly for neutral, happy, sad, angry, disgust, and contempt expressions; there was little between-expression synchrony, indeed these could often be asynchronous.

Intersubject correlations (ISCs) provided different information than measures of facial expression magnitude^7^^,^^11^ and avoid some issues relating to their analysis. Facial expression magnitude can tell us about how much each audience member might experience particular emotions during performances, as facial expressions are correlated with subjective emotional experiences.^62^ This type of measure could also in principle provide information on the degree of emotions experienced during discrete periods of performances. However, measuring expression magnitudes is dependent upon obtaining a baseline assessment of the degree of emotions expressed by each individual face when not experiencing the performance. Expressions would then need to be compared to this baseline, as faces can vary in terms of structure that resemble different emotional expressions.^63^^,^^64^ This approach would require all audience members to present an unexpressive or “neutral” face to the camera at a defined moment, not always possible when testing audiences “in the wild.” Furthermore, there can never be a guarantee that during any such baseline period audience members would not be experiencing any emotions that might impact their expressions. In contrast, expression ISC measures are not affected by this problem.

We tested how expression ISCs were related to predictions, made by the performance dramaturge, of the level of audience engagement during specific scenes of the performances. Performances were complex, involving multiple actors, scene changes, music, singing, and speech, and dealt with topics involving considerable emotional range. Predictions of how intensely these different elements of the performances might engage the audience consequently varied over the duration of the performances. However, predictions regarding periods of high engagement were not linked to a specific emotion or type of experience and could occur, for example, from periods of humor, jollity, or tragedy. Instead, the dramaturge’s scores were holistic assessments of the degree to which they might command the attention of audience members. In line with our hypothesis, we found that ISCs were positively correlated with predictions of audience engagement made by the performance dramaturge, in particular neutral, happy, anger, and disgust expression ISCs. The maximal correlation between expression ISCs and engagement in our study was seen with neutral expressions, reaching r = 0.40 for one performance, and average correlations between engagement and both neutral and happy ISCs were r = 0.32. Bayesian linear regression analyses showed that expression ISCs accounted for up to 24% of the variance in predicted audience engagement.

This relationship between facial expressions ISCs and predicted audience engagement is somewhat lower than a potentially comparable study examining neural synchrony and post-class ratings of engagement by students.^21^ Here, Dikker et al. measured EEG signals from 12 students during classes and calculated student-to-group synchrony while students rated the classes after the end of each class and at the end of the semester. They found that student-to-group synchrony was substantially positively correlated (r = 0.61) to the post-semester ratings. In a different study, Dmochowski et al.^22^ measured the relationship between neural synchrony and audience engagement and preference for television adverts. Neural synchrony was also measured with EEG from 16 individuals and was correlated with (r = 0.40) and explained only 16% of the variance in the engagement of a much larger and different group of individuals as measured by audience tweet frequency.

However, there are substantial differences between the methods we employ here compared with these other studies that may explain some of the differences in the magnitude of the relationship between synchrony and engagement. First, the signals we measure (facial expressions) are different from the neural signals recorded by Dikker et al.^21^ and Dmochowski et al.^22^ Second, the cognitive constructs underlying audience synchrony in our study are likely to be somewhat different. Dmochowski et al.^22^ localized sources of neural synchrony during advert viewing to large areas of cortex in both hemispheres stretching from sensory association areas in occipital cortex, along the superior temporal gyrus to the temporal poles, and within areas of parietal cortex. These areas include some regions involved in the experience of emotions.^65^^,^^66^ As such, our method captures in particular the affective responses to artistic performances, which are often a predominant feature of audience engagement,^3^^,^^12^^,^^13^^,^^67^ whereas these other techniques may capture additional cognitive interactions. Finally, our method shows that there appear to be independent synchrony profiles for the different emotional expressions, and these will be due to synchrony during different psychological processes. We saw greater synchrony of happy expressions in comparison with the other expressions measured. Although which expression was most synchronized varied through the performances, and this may reflect that different emotional processes synchronize differently during different cognitive appraisal of the performance.^42^

Any comparison with previous research measuring the relationship between the synchrony of different autonomic signals and audience engagement^17^^,^^18^ is somewhat harder due to the complex relationship between the production of expressions and the autonomic nervous system.^29^^,^^68^ However, most of these previously recorded signals (heart rate, respiration rate, skin conductance, etc.) are broadly dependent upon arousal level. Thus, there is perhaps some overlap between these measures and happy and anger expressions that tend to reflect high levels of arousal.^69^ Indeed, measures of heart rate and respiration rate synchrony appear related to concurrent measures of zygomaticus muscle synchrony in audiences.^18^

Furthermore, the measures of audience engagement in our study and prior studies^18^^,^^21^^,^^22^ are quite different. In contrast to these other studies, we used a prediction of audience engagement rather than a direct measure from the audience themselves. These predictions were based upon the dramaturge’s knowledge of the theatrical performances, including the historical context, purpose, and meaning behind the performances. As such, they represent a holistic estimate of many of the elements of the multifaceted concept of engagement^9^ including emotional responses, captivation, attention, and intellectual stimulation. Although these were predictions, and not direct measurements of engagement from the audiences themselves, what our method allowed was an estimate of audience engagement at different time points throughout the duration of the performances. This provides us with information to understand the relationship between the moment-to-moment fluctuations in audience expression synchrony, with our predictions of engagement at the same time points.

However, given that the measurement of audience engagement has proved difficult,^9^ measuring facial expression synchrony may provide a valuable method to non-invasively determine audience engagement during artistic performances as well as other group activities in the future. This technique has considerable benefits over other methods of measuring group synchrony. First, unlike other forms of measuring physiological (e.g., heart rate, respiration rate, skin conductance etc.) or neural (e.g., fMRI, EEG) activity, filming faces has little impact on the audience experience. There is no need to attach electrodes to the participants or interfere with the way they might experience the performance. Although our cameras were necessarily in view of the audience, they were often surreptitiously placed, and were likely quickly forgotten, so the audience experienced the performances in a naturalistic fashion and as intended by the directors. Currently, this is not achievable with any other technique.

Although not determined within this study, the synchrony of the different facial expressions within an audience may provide information about distinct aspects of audience engagement in the future (e.g., recall, change in attitudes, future attendance, etc.). The role of the neutral expression is currently uncertain but appears to be strongly linked to predictions of audience engagement and so clearly has potential utility for evaluating audiences non-invasively in the future. FaceReader classifies the intensity of the neutral and the other seven emotional expressions independently from each other. As such they provide different signals related to the emotional state of the audience members. The neutral expression does not represent the mere absence of all other emotional expressions, as it is only moderately negatively correlated with the other expressions within each individual. These findings might reflect that people can experience more than one emotion at the same time. This may be particularly the case when attending complex and varied artistic performances like ours where comic and tragic narratives were entwined. In contrast, more simple emotional experiences may result in more separable expressions.

Our methods allow the measurement of the simultaneous experience of the audiences as a group. This is not possible with fMRI, where ISCs are typically measured asynchronously.^25^^,^^61^ With other physiological signals (e.g., heart rate, skin conductance, etc.) simultaneous recording across the group is possible with multiple measurement systems but will be restricted by the availability of the equipment and to date has been restricted to low numbers of individuals.^10^^,^^21^ Furthermore, these methods are time-consuming to set up and invasive. In this study, we analyzed the simultaneous facial expressions of up to ∼50 individuals, which along with providing a perhaps more representative sample, enabled us to examine the dynamics of inter-individual synchrony. In principle, it would be possible to examine facial expression synchrony with even great numbers of individuals than those we measured here.

In alignment with our predictions, we found very strong evidence of a small effect of proximity on ISCs, where individuals closer to each other within the auditorium were more likely to show synchrony of neutral, happy, sad, angry, surprise, and fear expressions. One reason for the greater expression synchrony between individuals near to each other may be due to emotional contagion, as individuals in proximity have a tendency to “catch” other persons’ emotions, including their emotional appraisals, subjective feelings, patterned physiological response, and tendencies.^39^^,^^41^^,^^46^ Alternatively, this effect may reflect enhanced synchrony between friends who know each other sitting together in the auditorium. Typically individuals tend to forge friendships with others who share similar age, gender, and other demographic characteristics.^70^ Such similarities in personal characteristics can result in increased synchrony between individuals who are friends compared to individuals outside of their social network.^71^ This enhanced synchrony between nearby individuals may reflect either similar responses to the performance or enhanced emotional contagion due to proximity. Ultimately, these questions could be directly tested by comparing expression synchrony of individuals when alone to when they are sitting together as a group, either with strangers or with neighbors they know intimately.

The analysis of ISCs in large groups of individuals additionally allowed us to determine the impact of audience member age, empathy, and gender on ISCs. In line with our hypothesis, several (neutral, happy and angry) expressions were negatively correlated with age, whereas there was little evidence for a relationship for the other expressions. These results parallel those of others that have shown similar effects when measuring neural synchrony.^56^^,^^57^^,^^58^^,^^59^ Our results suggest that older audience members’ emotional experiences during the performances were more idiosyncratic, possibly resulting from this group’s poorer ability to recognize the emotions expressed by the performers.^72^^,^^73^^,^^74^

Empathy had a less substantial impact on ISCs, but as per our predictions, was positively correlated with neutral, happy, and angry expression ISCs.^21^ These results suggest that individuals showing higher empathy may experience these emotions alike during theatrical performances. However, our measure of audience member empathy was limited to completing a self-report questionnaire, for reasons of practicality. This method may not be optimal as individuals may not have the metacognitive insight to be able to accurately evaluate their own ability in this area.^75^^,^^76^ Therefore, it will take future testing with behaviorally verified measures of empathy to accurately measure the relationship between expression ISCs and participant empathy.

Due to the paucity of research on the role of gender in interindividual synchrony, we did not have strong predictions about the impact of gender on expression ISCs. Female audience members showed greater neutral, happy, and angry expression ISCs than males, indicating greater similarity in the emotions of female audience members. However, we had strong evidence that fear and contempt ISCs were the same across genders. These results stand in contrast to Petroni et al.,^58^ who saw greater ISC in males than females, but only in children and not in adults. We also saw no real evidence of an interaction between gender and age and so consider that these differences in our findings and Petroni et al.’s are likely due to the substantive differences in measures of facial emotional expressions and EEG signals.

### Limitations of the study

Measuring facial expressions in naturalistic environments is heavily dependent upon the lighting conditions that could be achieved and the constraints imposed by the physical space and the artistic demands of the performance directors. In earlier testing (e.g., in Madison in 2015 and 2016), our ability to record faces with video cameras at a high-enough quality to allow for face detection and expression extraction by our software was significantly compromised, and consequently we obtained very few faces on which to base our analyses. In contrast, when we have control over the positioning of the cameras and audience and the level of lighting (e.g., in the three performances in the Holbeck cinema, York 2017), we could ensure that faces were clearly visible to the cameras and were well illuminated. Here, we were able to obtain very good measures of facial expressions from the vast majority of the audience and through the entirety of the performances. As such, the use of this technique is limited to those situations where a predominantly full-frontal view of a well-lit face is possible.

Audience engagement was only positively correlated with neutral, happy, anger, and disgust expressions. This may perhaps be a product of the ability of FaceReader to classify these emotions well from our particular videos where faces were not filmed in optimal lighting conditions and videos were “up-sampled” by the digital zooming process. For example, classification of happy expressions relies heavily on detecting contraction of the zygomaticus muscle, which might be reliably detected with lower resolution information. In contrast, for example, classification of fear expressions relies on the detection of a combination of more subtle action units (e.g., inner brow raiser, upper lid raiser, lips part etc.) that might be harder to detect under the same conditions. Furthermore, FaceReader can confuse fear and surprise expressions.^77^ Consequently, the lack of relationship between engagement and some expressions may reflect the nature of filming multiple faces with each camera and the limitations of the version of FaceReader we used during our analysis. We will ultimately be able to test this possibility in the future with the use of many more cameras than we used here and as face expression classification technology improves.

In conclusion, we show that the synchrony of the facial expressions of audience members is related to predictions of audience engagement, as well as some of the characteristics of the audience members themselves. This technique could be used to measure the engagement of individuals at artistic performances and during other group activities. In comparison to previous measures of inter-individual neural and physiological synchrony, it is non-invasive and does not detract from the audience experience itself.

## STAR★Methods

### Key resources table

**Table:** 

REAGENT or RESOURCE	SOURCE	IDENTIFIER
**Deposited data**		

Raw data and statistical analyses	This study	https://osf.io/uzrhf/

**Software and algorithms**		

JASP	https://jasp-stats.org/	version 0.16.2
MATLAB	https://www.mathworks.com/	version 2020a

### Resource availability

#### Lead contact

Further information and requests for resources will be fulfilled by the lead contact, Nick Barraclough (nick.barraclough@york.ac.uk).

#### Materials availability

This study did not generate new unique reagents or other materials.

#### Data and code availability

• Raw data, secondary data and JASP statistical analyses have been deposited at the OSF and are publicly available as of the date of publication. The DOI is reported in the key resources table.
• This paper does not report original code.
• Any additional information required to reanalyze the data reported in this paper is available from the lead contact upon request.

### Experimental model and study participant details

#### Participants

Participants were audience members that attended one (or more) of 16 separate theatre and cabaret performances at different venues in the UK, USA and Australia, and consented to both have their face filmed during the performance and complete related questionnaires. A number of individuals attended these performances but did not consent to either filming or completing questionnaires. These additional individuals sat in seats in the auditorium out of the field of view of cameras. In total 455 participants were tested (130 male, 254 female, 71 other/prefer not to say; mean age = 39.9 years, SD = 20.8 years, range 15-86). Sample size calculations for the number of individuals required for analyses could not be carried out in advance as no suitable previous effect sizes were relevant. In addition, sample sizes for each performance could not be pre-specified as this was determined by the number of individuals prepared to fill in questionnaires and be filmed on the day of the performance. However, the samples used in our analyses are greater than any used previously that have measured neural or physiological ISCs. To ensure our measures of facial expressions were meaningful to the performance as a whole, rather than only a short segment of the performance, we only analyzed the expressions of those audience members whose facial expressions were possible to detect and model for a high degree (> 90%) of the time during the performance, and who had completed all questionnaires, and there was more than one individual that met these criteria in the audience (see Figure S2). These individuals attended one of 7 performances of “A comedy of us Jews” held in York, UK, 2017, or one of 3 performances of “Prince Bettliegend” held in Sydney, Australia, 2017. In total, our sample was 193 audience members (135 females, 58 males; mean age = 29.75, S.D. = 17.24, range 18 - 80). The study was approved by the ethics committees of the Departments of Psychology and Theatre, Film and Television at the University of York and local ethics committees where required, and were performed in accordance with the ethical standards laid down in the 1964 Declaration of Helsinki. Participants provided written informed consent for all testing.

### Method details

#### Theatrical and cabaret performances

Performances were as follows: “Laugh with us” by Lisa Peschel, based on a scripts by Felix Porges, Vítězslav “Pidla” Horpatzky, Pavel Stránský et al., performed in Madison, Wisconsin, USA, August 2015; “Harlequin in the ghetto” developed by a student ensemble based at the University of Louisiana Baton Rouge based on a script by Zdeněk Jelínek, led by Lisa Peschel and Alan Sikes, performed in Madison, Wisconsin, USA, May 2016; two different versions of “Harlequin in the ghetto” performed in succession on 2 separate nights in York, UK, June 2016 (on any one night audience members attended both performances in succession with an interval, however, audiences saw the performances in a different order on each day, and each performance is treated separately in analyses below); seven performances of “A comedy of us Jews” by Jac Weinstein, performed in 2 different venues in York, UK, June 2017; three performances of “Prince Bettliegend” developed by a professional cast at the University of Sydney, based on a script and lyrics by Josef Lustig and František Kovanic, led by Lisa Peschel and Ian Maxwell, performed in Sydney, New South Wales, Australia, August 2017 (during 2 of these performances each half of the audience were given differing introductory material for the purposes of research not reported here, each audience group in these two performances are treated as separate ‘performances’ in analyses below). All performances were part of a larger project, “Performing the Jewish Archive” where the intention was to perform previously neglected works by Jewish artists who had been displaced by various upheavals during the long twentieth century (ptja.leeds.ac.uk). The performances varied considerably and included monologues, dialogues and conversations, singing, comedy, pathos, and instrumental passages. Performances ranged from about 30 minutes to 1 hour in duration.

#### Filming audience facial expressions

Participant faces were filmed (1920 x 1080 pixels at 50 frames per second) with up to 4 HD cameras (3 x Panasonic HDC-TM900, 1 x Panasonic HDC-SD90) at each performance. Each camera was allowed to vary its white balance and focus automatically during the performance to help optimize images of the faces whilst the stage lighting varied during performances. Typically, cameras were situated unobtrusively on tripods or fixed to gantries at a height behind the actors so that approximately 15-20 faces were present in each camera’s field of view. As face expression analysis software relies on movies made under good light conditions,^78^ where possible, house lights were kept as bright as possible to help illuminate participant faces, although occasionally luminance was limited by the aesthetic concerns of the performance dramaturge. An additional camera, placed at the back of the auditorium, filmed the stage on which performances occurred.

#### Procedure

Testing was carried out in a range of auditoriums (see below table); typically, these were public or University venues used regularly for theatrical performances, and included a stage area and tiered seating. Auditoriums allowed for up to 100 seats, although not all seats were always taken at each performance.

**Table:** List of auditoriums where data were collected

Auditorium name	Address	Performance	Number of performances	Date
Promenade Hall, Overture Center for the Arts	University of Madison, Madison, WI, USA	Laugh with us	1	2015
Fredric March Play Circle, Memorial Union	University of Madison, Madison, WI, USA	Harlequin in the Ghetto	1	2016
BlackBox Theater	University of York, York, UK	Harlequin in the Ghetto	4	2016
Quaker Meeting House	Festival of Ideas, York, UK	A comedy of us Jews	4	2017
Holbeck Cinema	University of York, York, UK	A comedy of us Jews	3	2017
Seymour Center	University of Sydney, Sydney, NSW, Australia	Prince Bettliegend	3	2017

Participants were all paying members of the public (except for 3 performances of “A comedy of us Jews” performed in the Holbeck cinema in the Department of Theatre, Film and Television at the University of York UK in 2017 where all participants were paid £20 to attend). For paying members of the public, prior to purchase of tickets, participants were informed that the performance was for research purposes and that they could opt-in to this study by choosing to sit in specific seats on the day of the performance. When they arrived at the venue, information was provided to all audience members to indicate which seats were to be filmed and which were not so they could choose whether to take part in the study. Those audience members that wished to be participants in the study could choose to sit on those seats that were to be filmed, on which was also provided an envelope containing a pen, information sheet, consent form, and questionnaires. Each pack was labeled with a seat code, so that the participant self-reports could be linked to facial expressions in subsequent analyses.

Once all audience members had taken their seats, and prior to the start of the performance, a brief presentation explaining the study was given by one of the authors. Participants were asked to read the information sheet and sign the consent form. If they did not want to take part in the study they were informed that they could move to sit in an alternate seat at the venue. Before the performances, participants were given 10 minutes to complete a questionnaire to measure their degree of empathy (EQ-short: Madison 2015, 2016 & York 2016; QCAE: York 2017 & Sydney 2017) and an additional questionnaire to measure their current emotional state (data for an additional study not reported here). The EQ-short^79^ is a short form of the original Empathy Quotient^80^ developed to provide a fast self-report measure of empathy using only 22 questions. The EQ-short provides a measure of total empathy between 0 = no empathy and 44 = very high empathy. The QCAE is the Questionnaire of Cognitive and Affective Empathy^81^ and is a 31-item measure of empathy, but additionally can differentiate between cognitive empathy (e.g. perspective taking) and affective empathy (including emotional contagion and emotional and peripheral responsivity). The QCAE also provides a measure of total empathy between 4 = no empathy and 124 = very high empathy.

At the end of this period the cameras were turned on and the performance started. After the performance, cameras were turned off, and participants completed a questionnaire to measure their emotional state during the performance (data for an additional study not reported here), and to provide simple demographic information (age, gender). On completion of the final forms, participants placed all forms inside the envelope, sealed it, and placed the envelope within a box as they left the venue; following which they were offered a debriefing sheet.

### Quantification and statistical analysis

#### Analysis of facial expressions

To prepare the film for subsequent face expression analyses the film from each performance was edited (Adobe Premiere Pro CC; Adobe, San Jose, CA, USA). First, for each performance, films from all cameras were synchronized with each other by identifying the onset of a light marker flashed before the onset of the performance. We then generated separate movies of each participant face visible in each camera’s field of view. Where a face appeared in more than one camera field of view, we kept the movie that provided the most data (see below). The durations of face movies from an individual performance were identical, and lasted from the start of the performance (onset of acting) until the end of the performance (onset of audience clapping). Light intensity for the individual movies of faces from the 2015 and 2016 performances were increased using Adobe Premiere in order to help maximize subsequent detection of facial expressions. Lighting quality during 2017 performances was good enough so that movie light intensities were not adjusted. In addition, we ‘digitally zoomed’ into each face to upscale the video to 1920 x 1080 pixels for subsequent analysis.

Facial expressions were determined using automated face analysis software FaceReader 7.^82^ FaceReader works by fitting a model to the face present in the frame. However, it is not always possible to correctly fit this model, for example if the face is temporarily obscured by the participant’s hand or other object, the participant looks away from the front of the auditorium, etc. During frames when this occurred, it was not possible to derive information about any of that participant’s facial expressions. All faces were analysed using the software’s ‘general’ face model option unless data was available to indicate that the participant was 60 years old or over, where the ‘elderly’ face model option was applied.

Once a face is detected, FaceReader then applies two methods to determine what expressions are present. The first method synthesizes an artificial model of the participant’s face based upon approximately 500 key points on the surface of the face along with the facial texture of the area within these points. From a database of annotated images, the model calculates the main sources of variation in the face and then describes the deviations found in new images of the participant’s face from an average version of their face as a vector. Expression classification is done by inputting the vector into an artificial neural network. This network was trained on over 10,000 annotated images to classify seven emotional expressions and the neutral expression.

The second method of classification uses a deep artificial neural network to recognize patterns in the face. FaceReader directly classifies the facial expressions from image pixels, without generating any models; this helps FaceReader classify faces even when partially hidden. Finally, FaceReader combines these two methods to provide a classification of the 8 different expressions on a frame-by-frame basis. This classification is a measure of the intensity of each of 8 different expressions where 0 = expression not detected and 1 = expression fully present (see Figure S3 for an example output during a performance).

Faces of some individuals have a bias towards appearing to display certain emotions (e.g. anger) when in their resting (neutral) state. FaceReader 7 has the ability to be calibrated to adjust for this bias based on the participant’s neutral facial expression provided in a separate video of the participant’s face. For our earlier performances (2015, 2016) we used no calibration method. For our later performances (2017), we asked the audience before the performance to provide a neutral expression for a period of 15 seconds whilst looking directly at the cameras in front of them. From this neutral expression video FaceReader 7 was calibrated by taking the mid-point of the 15 seconds of the neutral expressions and running the calibration process on each participant’s face to control for these individual variations in physiognomy. The FaceReader option for automatic continuous calibration was not used.

#### Intersubject correlation (ISC) analyses

Intersubject correlations^26^^,^^61^ were calculated by correlating the expression intensity functions produced by FaceReader using MATLAB (The MathWorks, Natick, MA). Each expression of each individual audience member was correlated with the same expression in every other individual in the audience separately (i.e. happy-happy, sad-sad etc.). Series of correlations were calculated within a ‘sliding window’: a discrete sample of facial expressions over a period of 1 minute (3000 face images at 50 faces/s). Initially the window was positioned at the start of the performance, and measures of expression intensity from the first 3000 images of every face were correlated with measures of expression intensity from the first 3000 images from each of the other faces. Similar calculations were performed as the window was moved forward in time in 2 s jumps (100 face images) until the end of the performance was reached. Finally, r values calculated in each window from all face pairs were averaged separately for each expression, to provide measures of how synchronized the different audience expressions were at different points during the performance.

#### Audience engagement predictions

We restricted the analyses of how ISCs are related to predictions of audience engagement to 3 performances with large audiences (n = 44 to 50) that could contribute facial expression data for >90% of the whole performance in order to ensure a substantial number of ISCs (n = 946 to 1225) on which to base our analyses. In order to provide a prediction of the relative likelihood of audience engagement over the course of the performance, the dramaturge (author LP) provided a score for each scene of each performance by viewing the videos of the performance, without knowledge of the facial expressions of the audience members or the results of the ISC analyses. Scenes were defined to the nearest second and were interpreted as a change in action in the performance (e.g. character entering/exiting/doing something new). Scores were on a simple 1-3 Likert scale; 3 = high engagement, 2 = moderate engagement, 1= low engagement. These estimates (for an example see Table S3) were specific predictions of how strongly the different scenes will have commanded the audience member’s attention, based upon a range of different features of the performance including: the level of physical and verbal action on the stage, the level of emotion displayed by the actors, and how interesting or surprising certain moments of dialogue or action should be. High engagement was defined as the audience being ‘riveted’ during that section of the performance, following the performance both intellectually and emotionally very closely. Low engagement was defined as the audience likely not attending, having their mind wandering, or thinking about topics other than the performance. Whilst moderate engagement was defined as between these other definitions.

To allow a comparison with the expression ISCs, the dramaturge’s estimates of audience engagement during the performance were resampled by using the same sliding window method as for the ISC calculations. This had the effect of slightly smoothing the transitions between the 3 estimated levels of audience engagement. We then calculated the correlation between different expression ISCs and estimated audience engagement for each performance separately.

#### Statistical analyses

Before any statistical analyses were conducted, we examined the data for normality by plotting data distribution functions and Q-Q plots. We transformed all r and ISC values using Fisher’s r to z transform prior to statistical analysis.

In order to examine the influence of audience member spatial proximity on the synchrony of their facial expressions, we first calculated the Euclidian distance between all pairs of audience members. For simplicity we assumed that 1. the distance between adjacent seat positions on a row were equivalent to 1 unit, and 2. that the distance between similar numbered seats on adjacent rows was also equivalent to 1 unit. For statistical comparisons with ISCs, we used the square root of audience member distance to ensure that the data was normally distributed. We analyse the relationship between ISCs and proximity via Bayesian correlations and Bayesian regression. Although this analysis was conducted on transformed r to z values, and the square of the Euclidean distance between audience members, we additionally performed a permutation test to evaluate this analysis without assuming normality and homoscedasticity, and allow a more flexible assessment of the coefficients. We randomly permuted each of the expression ISC values and calculated the correlation coefficients between ISCs and distances; repeating this process 10,000 times to generate a null distribution. P-values for each expression were then calculated as the proportion of permutated correlations greater than or equal to the observed correlation.

Statistical analyses on ISCs were performed using Bayesian methods,^83^ using JASP.^84^ In all cases, a flat prior was specified as we had no prior published data that could inform our analyses and so we assigned a Cauchy prior distribution of $r=1/\sqrt{2}$ truncated to allow only positive effect size values. This study was not pre-registered, however, all analyses conducted are reported here.
