# Supplementary material for: Inter-subject correlation of audience facial expressions predicts audience engagement during theatrical performances
Source: iScience. 2024 Apr 29;27(6):109843. doi: 10.1016/j.isci.2024.109843 (PMC11109022; doi:10.1016/j.isci.2024.109843)
Supplement: Document S1. Figures S1–S3 and Tables S1–S3 [file mmc1.pdf]

iScience, Volume *27*

Supplemental information

Inter-subject correlation of audience facial expressions predicts audience engagement during theatrical performances

Richard A. Oakes, Lisa Peschel, and Nick E. Barraclough

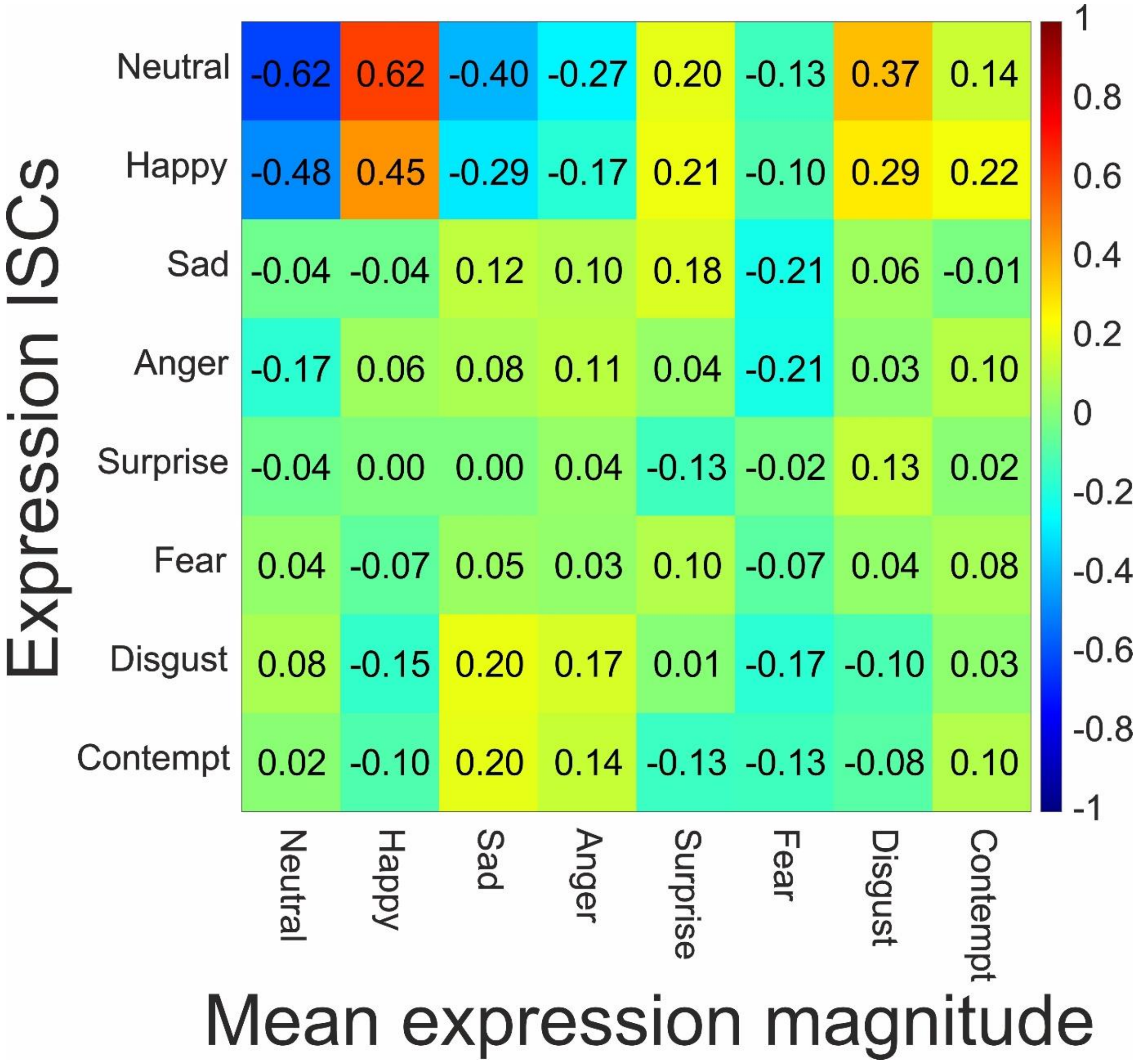

Figure S1. *Relationship between Expression ISCs and expression magnitudes.* Correlation matrix showing the correlation between mean expression ISCs and mean expression magnitudes. The matrix represents the average r values calculated across the 3 performances where ISCs were used to predict audience engagement.

Table S1.

Bayesian t-tests between on-diagonal vs off-diagonal r values.

Performance	BF_{10}	Error	Median posterior distribution	95% credible interval
P1	.38	.003	.222	[.010, .756]
P2	.28	<.001	.179	[.008, .659]
P3	.24	.001	.159	[.007, .612]

Note: Our alternative hypothesis was that ISCs were related to expression magnitude (on-diagonal > off-diagonal); our null hypothesis was that they were unrelated. Bayes factors show anecdotal (P1) or moderate (P2, P3) evidence for the null hypothesis.

Table S2.

Permutation test for the relationship between audience proximity and ISCs, related to Figure 3.

Expression	p
Neutral	<.0001
Happy	<.0001
Sad	<.0001
Anger	<.0001
Surprise	<.0001
Fear	<.0001
Disgust	.0020
Contempt	.0040

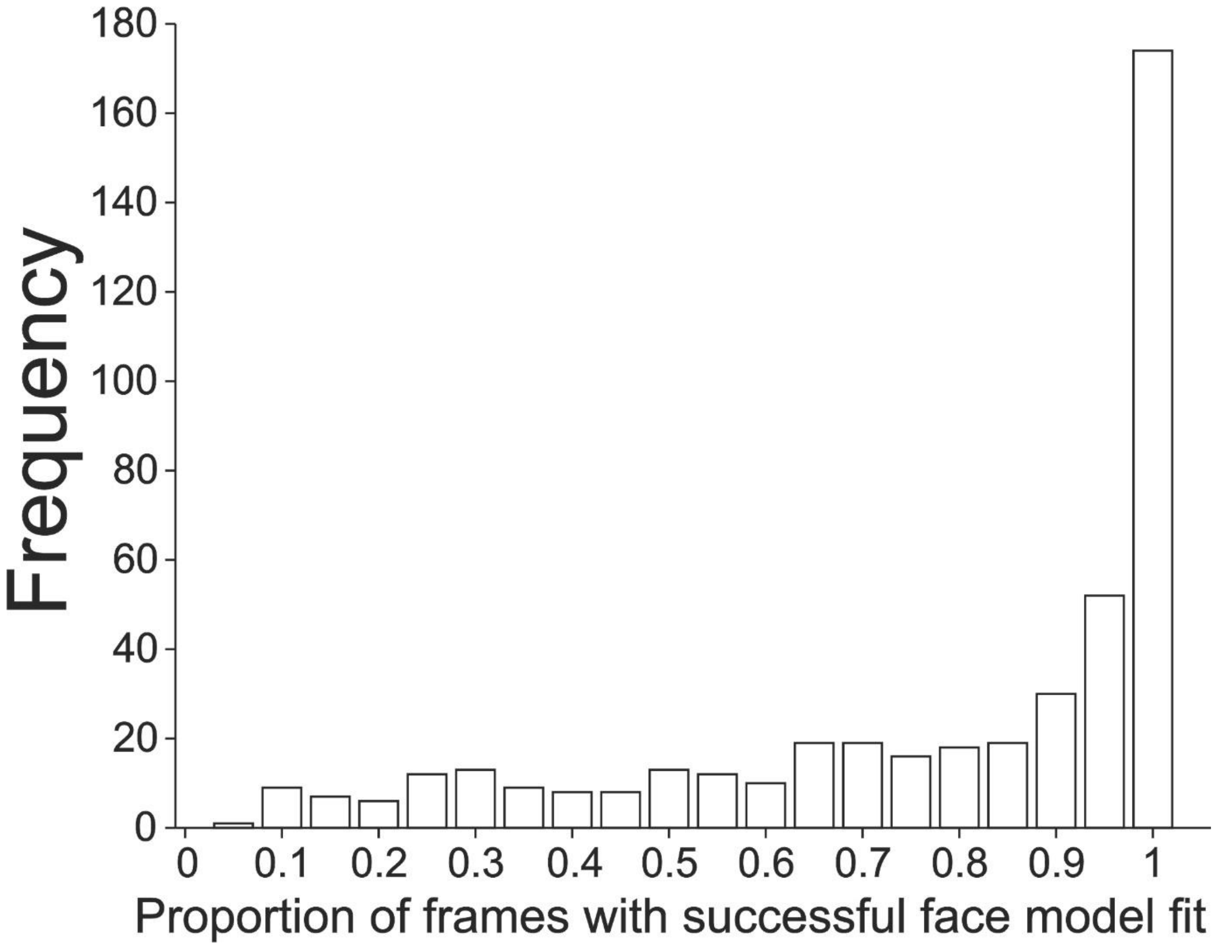

Figure S2. Face model fitting success, related to STAR methods. Frequency histogram indicates the number of participants where the face model can be fitted successfully. In total 455 faces were filmed, 193 met the criteria for testing (> 90% face detection + completing all questionnaires).

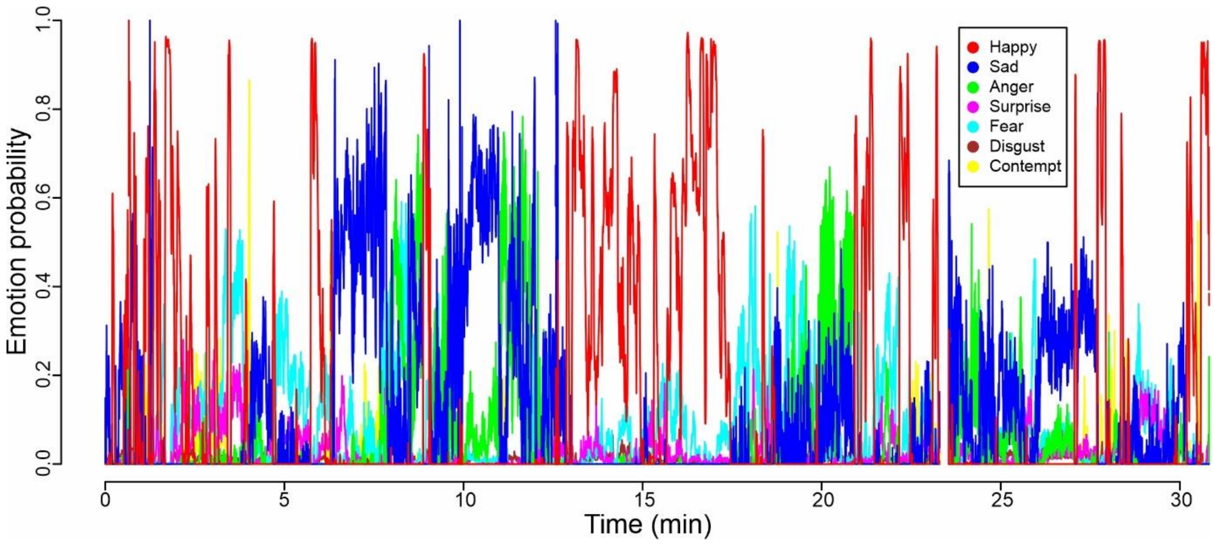

Figure S3. Extent and variance of 7 different emotional expressions, related to STAR methods. Lines indicate the likelihood functions of the presence of each emotional expression in a single individual over the duration of a performance in York 2017. The performance lasted approximately 32 minutes. X-axis shows the time course of facial expressions, when viewing the performance. Y-axis indicates the likelihood of each facial expression being detected, where 0 = not present, 1 = definitely present. Each coloured line indicates the different emotional expressions derived from the face (red-happy, blue-sad, green-anger, magenta-surprise, cyan-fear, brown-disgust, yellow-contempt). Gaps in the data for the expressions indicate periods when it was not possible to fit a model to the face.

Table S3. Example dramaturge engagement scores allocated to segments of a performance, related to STAR methods.

Start time of segment	Engagement score	Performance content
00:00:00	1	Show begins (actor's first line) 2+3 is 5 … clean the clothes
00:00:57	2	Women enter, fish for shabbes, why so angry?
00:02:20	1	Women leave, Joseph scolds David
00:02:37	2	Story of soldier killed in the war who lost his head
00:02:55	2	Customer enters, wants trousers, asst tries to sell him a coat
00:04:14	2	Customer tries on the coat, 'isn't it warm?'
00:04:45	2	'You could leave it to your son' Joseph enters, gives discount
00:05:20	2	David says there are not enough punches
00:05:38	3	Joseph starts shouting, kicks customer out
00:06:05	3	Joseph chases wife out, assistant sits at desk
00:06:25	2	Wife enters again, talks to David about suppliers' discount and bankruptcy
00:07:20	3	Wife: We don't have money? (crying theatrically, leaves)
00:07:53	3	David loses temper, MUSIC sings the Punches song
00:10:28	1	Joseph returns, end song, David leaves Joseph starts monologue about travel, Paris
00:11:22	2	Package delivery: mannequin
00:11:45	2	Mannequin unveiled, Joseph tips deliveryman
00:12:14	2	Mannequin tips over
00:12:22	3	Mannequin tips more flirtatiously
00:12:44	3	He sees mannequin come to life, she speaks French
00:13:21	2	Yes, I bought you at Lafayette,
00:13:38	2	I'm Joseph Kleiderman (shakes her hand)
00:14:07	3	Mannequin begins to approach him but gets jealous when he mentions Margaritte
00:14:46	3	He tickles her
00:14:53	3	BEGIN SONG wonder of wonders
00:15:36	3	She hugs him
00:15:45	3	Customer comes in for fur coat, Joseph gets rid of customer by saying they have nothing
00:16:13	3	Hannah comes in and accuses him of cheating
00:16:53	3	SONG Joseph Joseph
00:18:22	1	Phone rings, call about the bill, wife plays with mannequin then goes to phone
00:18:51	2	Wife returns to mannequin, who needs it?
00:19:22	2	Mannequin knocks her hat off
00:19:57	3	Mannequin pulls her hair, Hannah has a tantrum, leaves
00:20:20	2	Joseph and Mannequin flirt, she prepares to sing to him
00:20:31	2	SONG French song
00:22:09	3	SONG more active bridge
00:22:30	2	SONG final verse, embracing, kiss
00:23:11	2	She breaks away, he promises candles

00:23:46	2	Sexton enters, asks about pledge to the synagogue.
00:24:32	2	Joseph talks himself out of pledge
00:24:46	2	He denies he made a pledge
00:24:59	3	Story of Jew who wanted to cross the river
00:25:51	3	Pause, then they break out in laughter. Sexton leaves. Joseph: I earned £60 of candles
00:26:21	3	She is still, ‘Why is your hand so cold?’
00:26:48	2	David enters, Joseph orders him to wrap up the mannequin
00:26:59	3	Customer returns, ‘Here’s a ration card!’
00:27:20	3	Hannah returns, they sing ‘Hannah / Joseph’s’ song
00:28:17	3	All join in
00:28:27	3	Brief finale verse
00:28:38	2	Bows, applause
00:28:55	1	END
